# Increasing pathogenic germline variant diagnosis rates in precision medicine: current best practices and future opportunities

**DOI:** 10.1186/s40246-025-00811-z

**Published:** 2025-08-22

**Authors:** Sonam Dukda, Manoharan Kumar, Andrew Calcino, Ulf Schmitz, Matt A. Field

**Affiliations:** 1 Centre for Tropical Bioinformatics and Molecular Biology, College Science and Engineering, James Cook University, Cairns, QLD Australia; 2 Centenary Institute, The University of Sydney, Camperdown, Australia; 3 Computational Biomedicine Lab, James Cook University, Townsville, Australia; 4 Immunogenomics Lab, Garvan Institute of Medical Research, Darlinghurst, NSW Australia; 5 Menzies School of Health Research, Charles Darwin University, Darwin, Australia

## Abstract

The accurate diagnosis of pathogenic variants is essential for effective clinical decision making within precision medicine programs. Despite significant advances in both the quality and quantity of molecular patient data, diagnostic rates remain suboptimal for many inherited diseases. As such, prioritisation and identification of pathogenic disease-causing variants remains a complex and rapidly evolving field. This review explores the latest technological and computational options being used to increase genetic diagnosis rates in precision medicine programs.

While interpreting genetic variation via standards such as ACMG guidelines is increasingly being recognized as a gold standard approach, the underlying datasets and algorithms recommended are often slow to incorporate additional data types and methodologies. For example, new technological developments, particularly in single-cell and long-read sequencing, offer great opportunity to improve genetic diagnosis rates, however, how to best interpret and integrate increasingly complex multi-omics patient data remains unclear. Further, advances in artificial intelligence and machine learning applications in biomedical research offer enormous potential, however they require careful consideration and benchmarking given the clinical nature of the data. This review covers the current state of the art in available sequencing technologies, software methodologies for variant annotation/prioritisation, pedigree-based strategies and the potential role of machine learning applications. We describe a key set of design principles required for a modern multi-omic precision medicine framework that is robust, modular, secure, flexible, and scalable. Creating a next generation framework will ensure we realise the full potential of precision medicine into the future.

## Introduction

Identifying targetable disease-causing genetic variants lies at the heart of advancing precision medicine, improving clinical diagnostics, and enhancing our understanding of genetic contributions to diseases [1]. Precision medicine entails a healthcare delivery model that relies extensively on patient specific data points to guide the development of customised therapies [2]. One key driver of such initiatives is the ability to pinpoint pathogenic variants that greatly improves our mechanistic understanding of the disease process [3]. This progress has been largely enabled by the increasing affordability and accessibility of high-quality sequence data. Despite this progress, conclusively linking genetic variants with disease remains resource-intensive and time-consuming [4]. The most significant challenge remains differentiating the key genetic drivers from the large volumes of background genetic variation naturally present in every person. Further challenges exist with variant detection, annotation and prioritisation methods with the lack of global standards resulting in an over reliance on variable bespoke in-house solutions [5]. Groups such as the American College of Medical Genetics and Genomics (ACMG) are addressing this by providing guidelines on variant detection and interpretation; for example they propose guidelines to establish consistent cataloguing of genetic variants, classifying variants into five categories based on the strength of the evidence for disease causation. Despite these standards, many variants remain annotated as variants of uncertain significance (VUS), lacking sufficient functional assay data required for reliable classification [6]. Additionally, recurrent false positive variants can be included in clinical databases [7].

There is also an increasing recognition of the role of large and repetitive genetic variants in driving disease, however these remain challenging to detect with current short-read sequencing technologies and are better suited to more expensive long-read sequencing approaches [8]. Additionally, non-coding variants are being recognised in driving disease including intronic variants which create cryptic splice sites as well as variants modifying regulatory elements such as enhancers and promotors [9]. Another challenge specific to complex disease is the often-little understood interactions between genetic and environmental factors. Factors such as lifestyle and surrounding environment can contribute significantly to disease development and progression; however, our understanding of these processes is limited [10].

Segregation analysis of germline variants within families plays a critical role in precision medicine by enabling the accurate interpretation of genetic findings in the context of inherited disease risk [11]. By studying how a specific variant co-segregates with a disease phenotype across multiple family members, clinicians and researchers can distinguish pathogenic mutations from benign polymorphisms, thus improving diagnostic accuracy [12]. This analysis not only helps validate the clinical relevance of a variant but also informs risk assessment, surveillance strategies, and therapeutic decisions for both affected individuals and at-risk relatives. In precision medicine, where individualized care hinges on the precise understanding of genetic contributions to disease, segregation analysis remains a cornerstone for translating genomic data into meaningful clinical outcomes. Overall family data provides evidence needed to reclassify variants, either supporting pathogenicity through clear segregation or suggesting benign status through inconsistent patterns. This improved classification directly impacts clinical decision-making, from diagnosis and treatment selection to family planning [13]. Family analysis enables confident clinical recommendations, reduces uncertainty in genetic counselling, and identifies at-risk family members who would benefit from testing or enhanced surveillance, exemplifying the promise of precision medicine.

Despite steady progress, genetic diagnostic discovery rates for many complex diseases using traditional approaches remain low and capturing “missing heritability” requires multi-pronged approaches. These include a variety of sequencing-based (e.g. long-read, single-cell sequencing) and computer-based approaches (e.g. machine learning, multi-omic workflows). Robust frameworks capable of integrating huge volumes of complex patient data, genetic variant and annotation information are urgently needed [14].

## Sequencing technology

A diverse selection of sequencing technologies are now available for identifying genetic variants [15]. DNA-based solutions include whole genome sequencing (WGS), whole exome sequencing (WES), and targeted gene panels, while genetic variants can also be detected in cDNA used for RNA sequencing (RNA-Seq) [16]. Newer approaches include long-read sequencing, suitable for identifying more complex, larger genetic variants [17], and single-cell sequencing to identify rare or cell-type-specific variants [18, 19].

### Current DNA-based sequencing options

Targeted gene panel sequencing is appropriate when driver genes are largely known for a disease. In such cases, gene panels can obtain high diagnostic rates and the simple deployment, interpretation, and lower costs, offers an attractive alternative to WES/WGS [16, 20, 21]. Targeted panels are typically sequenced at a high depth to identify rare variants, a process which can be combined with unique molecular identifier (UMI)-based approaches to further increase resolution [22]. There are limitations of this approach however such as their inability to identify novel variants and large genetic variants [16, 23].

WES is effective in finding both known [24] and novel driver mutations [25] in coding regions of the genome. WES employs a targeted approach via a capture array containing most known coding exons, thus covering the majority of the coding regions [3]. Despite only accounting for ~ 1% of the genome, an estimated 85% of mutations responsible for diseases are thought to occur within exons [26–28]. WES is attractive with regard to price and sequence depth relative to WGS [29, 30].

WGS is an unbiased method that provides sequence data across the entire genome [31]. WGS is increasingly becoming the first choice for patient sequencing due to advantages including the ability to detect small and large genetic variants as well as achieving relatively even sequence coverage [32–34]. The diagnostic superiority of WGS to chromosomal microarray (CMA), karyotyping, targeted sequencing assays and WES [35–40] has been demonstrated. Accordingly, precision medicine programs are employing WGS as the first option resulting in the development of increasingly standardised methodologies [41, 42].

### Current RNA-based sequencing options

Bulk RNA-seq is a high-throughput method used to examine the complete set of RNA transcripts within a biological sample [6]. Bulk RNA-Seq typically obtains sequence data from a mixed heterogenous population of cells in contrast to tagged individual cells as is done in single-cell RNA-Seq (scRNA-Seq) [43]. The clinical utility has been demonstrated largely for the ability to identify dysregulated genes that warrant further investigation within the genome [44]. Additionally, RNA-Seq allows the identification of aberrant splicing events such as retained introns or skipped exons and gene fusions.

### Single-cell sequencing

In contrast to traditional bulk sequencing methods, single-cell technologies incorporate cell-specific barcodes to obtain per-cell sequence information for thousands of cells simultaneously. To date, most single-cell platforms utilise RNA as input (scRNA-Seq) however a growing number of platforms offer single-cell DNA sequencing (scDNA-Seq).

Single-cell RNA sequencing is an advanced technology able to evaluate transcriptional similarities and variances within a population of cells, revealing cell-type-specific levels of heterogeneity previously undetectable by bulk sequencing methods [45–47]. Nonetheless, scRNA-Seq remains technically challenging with limitations including generation of doublets and dead cells, lower sequencing depth per cell, data sparsity, high input cell requirements, and high cost. Despite these challenges, scRNA-Seq offers an unprecedented opportunity to track disease progression within heterogenous cell populations.

Single-cell DNA sequencing allows the detection of per-cell or cell-type-specific rare genetic variants from mixed heterogenous input samples [48]. Many of the same limitations are shared with scRNA-Seq, however variant detection is feasible for targeted gene panels using technologies such as Mission Bio’s Tapestri platform. Additionally, significant amplification is typically required [49], a process known to introduce errors and uneven coverage resulting in challenges in downstream data analysis [50].

### Long-read sequencing

Third generation single molecule long-read sequencing overcomes many of the limitations of short-read technologies [51]. Initially plagued by high error rates, continual improvements are producing progressively longer and higher quality reads, with lengths of up to 2 Megabase pairs now possible [52, 53]. The third-generation sequencing market is primarily dominated by two technologies: (i) Pacific Biosciences and (ii) Oxford Nanopore Technologies (ONT) [54], both of which offer DNA or RNA based sequencing. Long-read DNA sequencing is critical if driver variants are repetitive or complex in nature (e.g. long tandem repeats, copy number variants) or occur in repetitive gene families, GC-rich regions or pseudogenes. Long-read RNA sequencing captures full-length isoforms and can identify novel transcripts, skipped exons, retained introns and gene fusion products [55].

## Prioritisation strategies

**Fig. 1:**
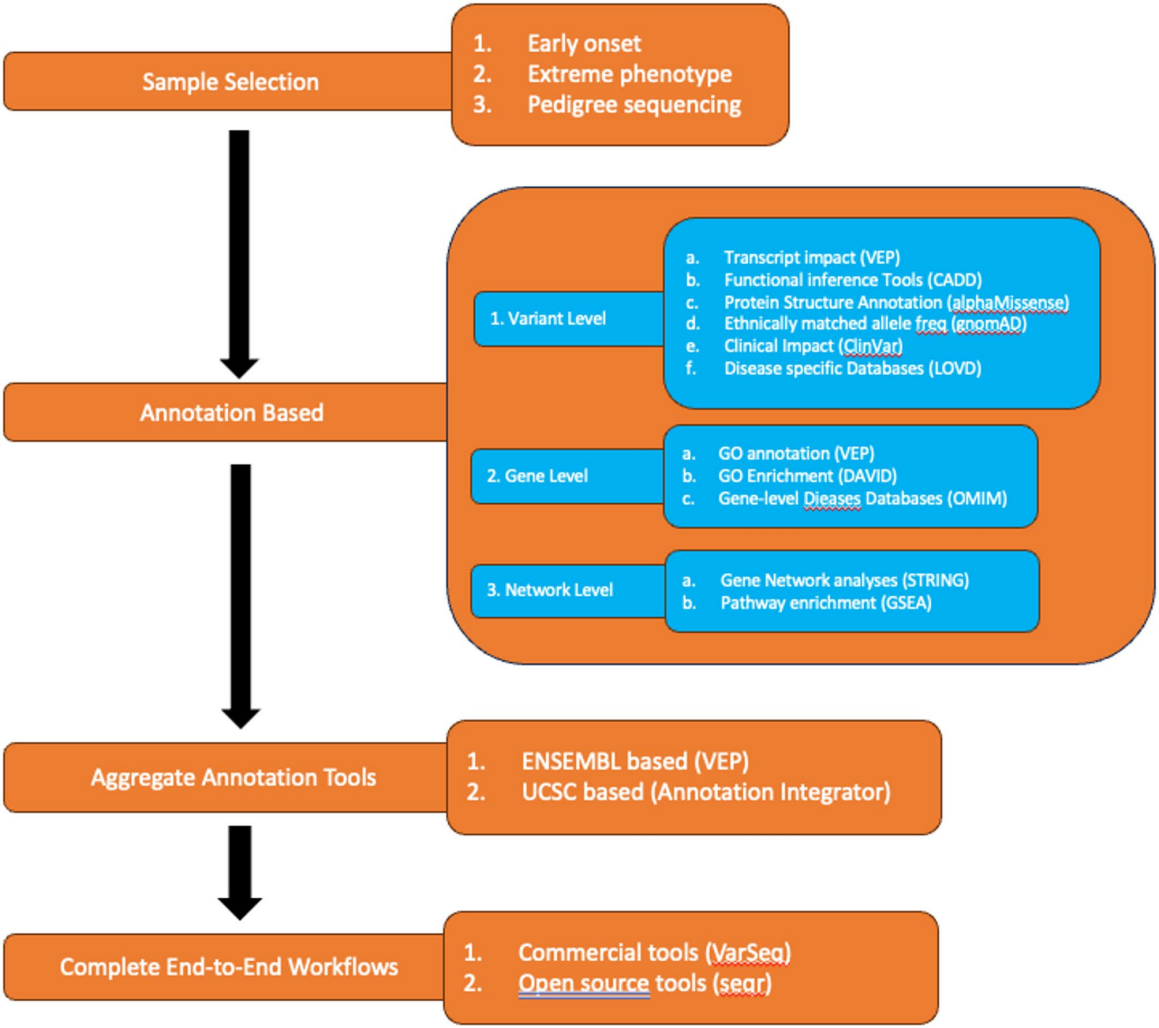
Variant prioritisation strategies

All sequencing approaches generate large numbers of genetic variants, the majority of which are not relevant for the underlying disease. Reducing the genetic search space for causal variants can be done through a combination of careful patient selection, variant annotation (at the level of variant, gene and gene network), and software development and optimisation (Fig. 1).

### Sample selection strategies

Sample selection strategies are critical to increasing the likelihood of identifying disease causing variants. Common strategies include grouping unrelated individuals by phenotype, selecting patients with early onset and/or extreme phenotype and pedigree sequencing for families.

For single or unrelated individuals, strategies include sequencing samples with early onset or extreme phenotypes as well grouping patients with similar phenotypes that potentially share an underlying genetic cause [56]. Choosing unrelated individuals with a shared well-characterised phenotype requires annotation with standardised human phenotype ontology (HPO) terms [57]. Further defining inclusion and exclusion criteria based on clinical features - disease progression, or other relevant parameters – allows the generation of a study cohort that is relatively homogenous and well suited for detecting shared driver variants (or pathways) linked to the observed phenotype [58]. The significance of careful patient selection is highlighted in numerous studies as an effective way to increase diagnosis rates [59, 60].

### Pedigree-based strategies

For related individuals, utilization of pedigree sequencing is an extremely effective strategy for reducing the genomic search space for causal variants. Pedigree sequencing is particularly useful for the identification of rare familial variants which segregate with the phenotype of interest [61, 62]. This approach yields additional information including inheritance modes and can track the segregation of variants within families, however custom software is required [12]. Sequencing of a proband child with healthy parents is very successful for rare, early onset diseases by focusing on a small number *de novo* variants. Similarly, consanguineous pedigree sequencing reduces the search space to homozygous variants [63].

Segregation analysis plays a vital role in interpreting and prioritising genetic variants by examining how a variant is inherited within a family and whether it consistently appears in affected individuals while being absent in those unaffected. Such co-segregation patterns strengthens the case for pathogenicity, particularly when it is aligned with the expected pattern of inheritance [64, 65]. In the line with ACMG/AMP guidelines, segregation data can count towards variant classification as ‘supporting evidence (PP1)’, with increasing weight assigned as more segregation evidence is observed [66]. Recognition of segregating *de novo* or very rare variants in dominant conditions further strengthens the case for pathogenicity (PS2) [65]. In contrast, variants that do not segregate with disease phenotype in affected family members may indicate benign status, reduced penetrance, or the presence of phenocopies—individuals who exhibit symptoms due to non-genetic factors or different underlying genetic causes [67]. Such scenarios may point to potential locus heterogeneity, where similar phenotypes arise from mutations in different genes [68, 69]. The identification of such cases is vital for accurate genetic diagnosis. When causal variants are present in asymptomatic carriers, we can estimate penetrance and variability in disease severity thus enabling personalized monitoring strategies and more accurate prognoses for at-risk relatives [70].

### Annotation-based strategies – variant level

Variants are first annotated based on the exact genomic coordinate and the observed nucleotide change. This includes assessing impact on transcripts, estimating impact on protein function, comparing to population variant levels and overlapping with clinical variant databases.

Variants are typically overlapped to a transcript model and stratified according to their impact using popular tools such as SnpEff, VEP or ANNOVAR [71–73]. High level genomic overlaps are first considered (e.g. inter-genic, intronic, exonic, etc.) and further refined by transcript effect when appropriate (e.g. exonic SNVs classified as synonymous or non-synonymous, exonic indels classified as frameshift or non-frameshift). Next, variants are run through functional inference tools to predict potential functional consequence of genetic variants. These tools utilise information including evolutionary conservation patterns, protein annotations and structural information to make predictions about the functional impact of genetic variants. Many tools exist including PolyPhen2 and SIFT [74, 75] for missense mutations and CADD for all variant types [76]. While these tools are useful, their results should be combined with other types of evidence as they are known to suffer from high false positive rates, particularly for variant subclasses such as pharmacogenetic variants [77, 78].

Until recently, a significant limitation in the annotation of protein features was the lack of comprehensive protein structures for all human proteins. AlphaFold successfully generated extremely accurate protein structures for all human proteins offering new opportunities for variant prioritisation [79]. The accuracy of functional impact tools can be increased with AlphaFold integration by considering the structural context of genetic variants [79]. AlphaMissense was subsequently developed based on AlphaFold2 predictions and fine-tuned using human and primate variant frequency databases [80]. Over time AlphaMissense (and subsequent developments) are likely to play a big role in improving functional impact metrics [81]. Overall there are many options for variant functional annotation (e.g. ENSEMBL Variant Effect Predictor (VEP), ANNOVAR, SnpEff) and functional inference prediction (Polyphen2, SIFT, CADD, AlphaMissense). Table 1 summarises several options including their relative strengths and weaknesses.

**Table 1:** Variant annotation tools

Software (Class)	Strengths	Weaknesses
VEP (Functional annotation)	Open source; supports web, CLI, and API usage; annotates coding/non-coding variants via Ensembl & RefSeq models; integrates allele frequencies, pathogenicity scores & phenotype databases; customizable output & filtering options; regularly updated.	Complex output with multiple transcript annotations; requires filtering to simplify; non-coding annotations require additional configuration; plugin setup can be complex; slower performance on large datasets without caching.
ANNOVAR (Functional annotation)	Broad annotation support (e.g., RefSeq, gnomAD, CADD); flexible framework; efficient variant filtering; fast runtime.	Lower HGVS accuracy (93.3% concordance); limited support for complex/structural variants; requires manual database updates; not optimised for polygenic traits; collapses transcript isoforms.
SnpEff (Functional annotation)	Fast annotation for high-throughput pipelines; supports multiple genomes & transcript models; Coding annotation accuracy (~ 89.8%) comparable to VEP.	Lower concordance for indels & frameshifts (< 75%); protein syntax often mismatches references;
Polyphen2 (Functional inference prediction)	Predicts impact of missense mutations based on protein structure and evolutionary conservation.	High false-positive rate for certain variant classes, does not handle non-missense variants.
SIFT (Functional inference prediction)	Missense variants prediction based on sequence conservation.	Lower accuracy for less conserved regions of proteins.
CADD (Functional inference prediction)	Combines 60 + annotations into a single impact score; ranks deleteriousness across coding & non-coding variants; Incorporates both simulated & observed variants for robust training; Machine learning (SVM) framework improves generalizability and prioritization.	Lacks variant-type specificity (e.g. splicing vs. missense); less precise for rare or population-specific variants ; computationally demanding for non-pre-computed variants; may inflate scores for non-coding variants.
AlphaMissense (Functional inference prediction)	Combines structure & conservation; high concordance with REVEL/CADD; effective for prioritizing pathogenic missense variants.	Still emerging; may inflate pathogenicity scores in some domains; needs further validation; accuracy varies across genes/proteins classes.

Population level databases of variant frequency are a powerful tool for understanding potential biological impact of genetic variation both globally and within matched ethnicities. Assigning variant allele frequencies enables the identification of rare or novel variants, a group enriched for pathogenic variants [82]. Variant databases have grown progressively larger over time, however historically most variants were of European origin [82]. Databases such as dbSNP and 1000 Genomes are two of the earliest databases and have proved invaluable for assigning variant frequencies [83, 84]. The Genome Aggregation Database, commonly known as gnomAD, is a more recent entry providing a comprehensive and publicly accessible repository that aggregates genomic data from a diverse range of populations [85]. It provides a wealth of information regarding the frequency and distribution of genetic variants across the human genome [86]. Critically, population databases have recognised the importance of incorporating non-European individuals, however many groups remain underrepresented [87].

Disease focused variant databases are another critical tool for variant prioritisation. While population level databases serve to filter out large numbers of common variants, disease variant databases help identify candidate pathogenic variants. These databases contribute significantly to the interpretation of genetic variants in a clinical context, aiding in the identification of variants associated with diseases thus informing clinical decision-making. Some of the larger databases include ClinVar [88], Human Gene Mutation Database (HGMD) [89] and Leiden Open Variation Database (LOVD) [90]. ClinVar is one of the largest clinical genomic databases, serving as a repository for variant data from clinical laboratories, clinicians, expert groups, patients, researchers, and other databases; it is a freely accessible, publicly curated database maintained by the National Centre for Biotechnology Information (NCBI) [91]. ClinVar ranks variants based on evidence such as functional assays providing a consistent scoring system across all potential clinically relevant variants. Similarly, the Human Gene Mutation Database (HGMD) aims to catalogue all mutations associated with inherited diseases [92, 93]. The mutation data in HGMD are sourced solely from scientific literature and undergo rigorous manual curation using manual screening of journals and automated text mining [93]. Finally, LOVD is a freely available web-based platform for the collection, display, and curation of DNA variants in locus-specific databases (LSDBs) [90]. The design of LOVD system includes flexibility and seamless integration with other locus-specific LOVD instances, and introduced the “custom column” feature, enabling curators to tailor field setups according to their needs [94].

### Annotation based strategies – gene level

A critical decision in any annotation workflow is the selection of the gene model. There are many efforts to standardise both gene sets and naming conventions however many challenges persist. Some of the popular models include ENSEMBL [95], GENCODE [96], RefSeq [97], UCSC [98] and UniProt [99]. Table 2 highlights the models’ strengths and weaknesses.

**Table 2:** Gene model options

Gene Model	Strengths	Weaknesses
ENSEMBL	Reliable cross-species gene annotation via combined manual & automated methods; supports transcript diversity & comparative genomics; regularly updated with VEP & BioMart links; core genome browser support.	Annotation varies by species; complex transcripts could be inconsistently modelled; dependent on quality of assembly genome
GENCODE	High-quality gene annotation for human/mouse; includes lncRNAs, pseudogenes, & transcripts; integrates manual curation & automation; captures transcript diversity; used within Ensembl, RefSeq, & UCSC	Incomplete experimental support for all transcripts; redundancy & unclear function in many lncRNAs / pseudogenes; manual curation limits scalability; inter-version differences may affect coordinate tracking.
RefSeq	High-quality, curated reference sequences for genomic, transcript, & protein data; consistent across species annotations; integrate manual curation with scalable automation; widely adopted in tools like VEP, ANNOVAR, GATK.	RefSeq tends to be conservative and includes fewer transcript isoforms; RefSeq updates less frequently than other models; RefSeq is centrally managed by NCBI and no community input
UCSC	Curated gene models from mRNA/protein alignments enhances RNA-seq quantification; emphasizes reliable transcripts & simplifies isoform sets for reproducible gene counts; integrated with UCSC Genome Browser.	Limited transcript diversity & isoforms & non-coding RNAs. Fewer splice junctions reduce RNA-seq accuracy; biased toward canonical genes
Uniprot	Provides detailed protein-level annotation including domains, function, and subcellular localization	Does not directly annotate variants or regulatory elements; protein-focused

In addition to variant-specific annotations, gene-level annotations are subsequently applied. In many cases a specific variant may not have been explicitly linked to disease pathogenesis, however the role the gene plays in driving the disease is well characterised. Gene level annotations largely consist of disease databases and gene ontology (GO) term annotation and enrichment analysis. Databases are typically curated repositories of gene-disease associations and help identify whether gene dysfunction has previously been implicated in similar diseases. There are many such databases, the largest being the Online Mendelian Inheritance in Man (OMIM) database [100].

If, however, a gene has not been directly implicated in causing the disease, gene ontology (GO) is able to identify the function for each gene of interest potentially linking GO terms to the observed disease phenotype. GO annotation is standardised through large international efforts such the Gene Ontology Consortium (http://www.geneontology.org) which enable quick and easy GO annotation of structured domain-specific ontologies [101]. A common application using GO terms is enrichment analysis, which aims to identify over-represented biological process, molecular function or cellular component shared by genes implicated in driving a polygenic disease.

### Annotation based strategies – gene network level

Beyond gene-level annotations, the gene’s role within larger biological networks can be examined. To do this there are a variety of gene network analysis tools designed to analyse and interpret biological data, particularly gene expression data, in the context of biological networks. These tools aim to uncover relationships and interactions between genes to gain insights into the underlying biological processes. They contribute significantly to the understanding of the complex relationships within biological systems, helping researchers unravel the functional implications of gene interactions. Several popular tools in this space are STRING and Ingenuity Pathway Analysis (IPA). STRING (Search Tool for the Retrieval of Interacting Genes/Proteins) is an online platform created to identify protein-protein interactions (PPIs) and functional associations [102]. The STRING database, available at https://string-db.org/, systematically compiles and integrates protein–protein interactions, encompassing both physical interactions and functional associations. The database is populated from various sources, including automated text mining of scientific literature, computational predictions based on co-expression and conserved genomic context, databases containing interaction experiments, and curated sources describing known complexes and pathways [103]. IPA is a proprietary tool developed by QIAGEN with similar functionality that is employed for applications including biomarker discovery, metabolomics, microRNA research, next-generation sequencing data analysis, proteomics, toxicogenomics, and transcriptomics [104]. While most current gene network tools incorporate well-characterised large, curated datasets new tools that construct custom networks from genome/transcriptome patient data for a single patient offer potential for custom treatments [105].

### Comprehensive annotation tools

While many pipelines overlap annotation datasets consecutively in series, aggregate tools are becoming increasing popular to manage the increasing number of disparate annotation resources. With continuously updated databases, it is increasingly important to apply consistent, up to date annotations [106]. ENSEMBL’s Variant Effect Predictor (VEP) is a prominent aggregate tool in the landscape of functional annotation. VEP provides comprehensive annotations for genetic variants, including their functional consequences, conservation scores, and potential associations with known diseases [107]. The tool is known for its user-friendly interface and frequent updates, ensuring that researchers have access to the latest genomic information by linking results to ENSEMBL’s latest gene model. VEP’s ability to handle diverse types of genomic variants and its integration with various databases make it a valuable resource in annotation improvement efforts [108]. Additional tools like ANNOVAR perform a similar role [109].

### Complete end-to-end workflows

Beyond aggregate annotation tools, there are an increasing number of complete end-to-end variant prioritisation workflows. These tools typically integrate functionalities including variant annotation, filtering, and interpretation aiming to identify potentially pathogenic variants directly from input variant lists with little to no manual interpretation required. Example include VarSeq, a commercial variant analysis software tool that is designed to streamline the entire workflow, from variant discovery to interpretation for gene panels, exomes or genomes [110]. Non-commercial options include WANNOVAR, a web-based tool designed for annotation and functional prediction of genetic variants and VariantDB which is designed for the annotation, prioritisation and analysis of genetic variants [111]. Seqr is an increasingly popular option developed by the Broad Institute [112].

While this next generation of tools are promising, challenges with installation and lack of configurability hamper their widespread uptake with researchers and clinicians often favouring to combine multiple tools to perform concurrent steps in bespoke workflows [38].

## Machine learning applications

Addressing the significant challenges associated with pathogenic variant identification requires a multi-faceted approach. One potential solution being considered is the development of advanced machine learning (ML) algorithms. By leveraging ML, algorithms can potentially improve diagnosis rates by identifying underlying complex relationships within biological systems [113]. ML algorithms are gaining traction in life sciences due to the capacity to deal efficiently with complex genomic data patterns [114]. Early works suggest that ML algorithms have the potential to learn from, and act upon complex heterogeneous datasets by identifying new biological patterns that increase diagnosis accuracy [115–118]. It is likely that ML algorithms will play an increasingly important role in detecting pathogenic variants. Some recently published ML algorithms for precision medicine are listed in Table 3 .

**Table 3:** ML applications in precision medicine

Software	Function	ML approach	Website
AlphaMissense	Functional inference	Deep learning	https://alphamissense.hegelab.org/
DeepVariant	Variant detection	CNN	https://github.com/google/deepvariant
M-CAP	Variant prioritisation	Supervised ML	http://bejerano.stanford.edu/MCAP/
MLVar	Variant prioritisation	Method/Pipeline using ML	https://github.com/GiovannaNicora/MLVar
REVEL	Functional inference	Random Forest (RF)	https://sites.google.com/site/revelgenomics/
BayesDel/ PEARCH	Functional inference	Likelihood Based approach	https://fenglab.chpc.utah.edu/BayesDel.html
ClinPred	Functional inference	Random forest and Gradient boosting models	https://sites.google.com/site/clinpred/
MAVERICK	Functional inference	Neural network-based	https://github.com/ZuchnerLab/Maverick
EvAgg	Clinical curation	LLM model	https://github.com/microsoft/healthfutures-evagg

### Variant pathogenicity

One of the most common current applications of ML is predicting variant pathogenicity, with numerous computational tools available [119–129]. ML models are typically trained on both known pathogenic and benign variants and deployed to predict the functional consequences of genetic variants on both protein structure and function [130]. Depending on the composition of training data, these approaches can be divided into genome-wide, disease-specific, or even gene-specific categories [131]. Popular tools offering genome-wide data predictions include Rare Exome Variant Ensemble Learner (REVEL) [124], BayesDel [132], ClinPred [133] and AlphaMissense [52].

### Variant prioritisation

Other common ML applications include variant detection, prioritisation and feature discovery. DeepVariant is an increasingly popular variant calling workflow compatible with Illumina, PacBio HiFi, and Oxford Nanopore sequence data [134]. DeepTrio is built upon DeepVariant and uses neural networks to identify variants specifically in pedigree of two or three members. M-CAP is a prioritisation tool that eliminates uncertain variants and reports 95% sensitivity levels [130]. MLVar is a another variant prioritisation workflow that follows ACMG guidelines and uses variant annotation features to predict probabilistic pathogenicity scores [135]. MAVERICK uses a neural network approach to predict pathogenic variants for Mendelian monogenic diseases [136]. Identifying cryptic biological features is another important ML application, for example the prediction and recognition of transcription start sites (TSSs) [137], splice sites [138], promoters [139], enhancers [140], and nucleosome position [141]. Larger end-to-end workflows employing ML are an active area of development.

Genome-wide association studies (GWAS) are another active area of ML algorithm development. Tools such as genomic best linear unbiased prediction (gBLUP) [142], support vector machine (SVM) [143], xGBoost [144], and random forest (RF) [145] are widely used to identify relevant traits in GWAS, with the large, well annotated GWAS data suitable for training purposes. Similarly, large datasets divided into disease and controls serve as suitable training data for ML algorithms that are able to predict traits and identify enriched variants [146]. Many tools however fail to generalise to specific diseases. To address this, studies often run multiple ML methods using a consensus-based approach.

### Large Language model

Large language models (LLM) show promise in a variety of precision medicine applications such as reducing literature search time for variant classification and interpretation. Microsoft developed the generative AI tool the EvAgg, which reports improvements in the sensitivity and specificity of pathogenic variant identification [147]. EvAgg reduced the manual curation time by 34% and increased the number of papers, variants, and cases evaluated per unit time. Such works have led to papers concluding that variant classification will be standardized in the near future, however achieving this requires overcoming significant challenges [148]. A recent benchmarking study considered four LLMs across ten fictional oncology patients and encouragingly found that LLMs were able to identify several important treatment strategies and provide some reasonable suggestions not easily found by experts [149]. However, they concluded that LLMs are not yet applicable for routine clinical analysis as an aid for clinical decision-making in oncology. Analysing complex germline disease represents an even bigger challenge.

While ML holds great promise for precision medicine, it is not without challenges due to the complexity and uniqueness of an individual’s genome highlighting the need for accurate, robust and interpretable models. Challenges include issues with the accuracy of existing variant classifications as well as the rising number of variants of uncertain significance (VUS) [150]. Building robust ML models requires large, high-quality data that has been extensively benchmarked using both simulated and established reference data sets [151]. Generating such data sets is compounded by the inherent complexity of the human genome, with numerous non-genetic factors contributing to complex disease. While ML algorithms are increasingly able to process complex genomic information to identify novel patterns and associations relevant to variant interpretation, patient datasets are increasingly heterogeneous in terms of data type and source [152]. Increasingly, data sets contain a number of data modalities (e.g. transcriptomics, epigenomics, and clinical data) requiring updates and changes to existing ML models trained exclusively on other types of data [153]. ML algorithms face additional challenges with their need for large diverse and homogenous training datasets without potential biases, a known challenge with many complex molecular datasets [154]. Overall, the most significant barriers to widespread clinical adoption of ML approaches are model interpretability, model validation and data harmonisation.

Model interpretability remains one of the most significant barriers to clinical uptake as any critical treatment decisions require a clear understanding of how models arrive at recommendations. Deep learning models, while highly effective at pattern recognition in genomic data and medical imaging, often function as “black boxes” where the decision-making process remains opaque. This is particularly problematic in precision medicine, where treatment decisions involve weighing complex risk-benefit profiles for individual patients. For example, a neural network might accurately predict cancer treatment response, but if clinicians cannot understand the biological rationale, they will be reluctant to act on it. New tools such as StratoMod [155] are using interpretable ML to predict variant calling and sequencing errors however without additional orthogonal validation data, clinical uptake remains unlikely. To address ‘black box’ challenges in security sensitive applications new tools like EnEXP are using interpretable ensemble tree approach to achieve a global interpretation of the entire dataset through the aggregation of individual sample insights [156]. Recent advances in explainable AI, including LIME and SHAP, offer promising approaches, but these post-hoc explanations may not accurately reflect the model’s true decision-making process [157].

Data validation is limiting clinical uptake as it presents unique challenges beyond traditional evaluation metrics. While models might demonstrate excellent performance on test sets, real-world clinical translation requires additional considerations. Temporal validation represents a particular challenge as medical practice and treatment protocols evolve continuously [158], for example models trained on historical data may not perform reliably on current patients in rapidly advancing fields like oncology where new biomarkers are regularly discovered. External validation across different healthcare systems and patient populations is also essential but difficult to achieve in practice. Models developed at academic centres may not generalize to community hospitals with different demographics. Overall, the validation process must account for clinical decision-making’s dynamic nature, where model predictions influence subsequent patient management, creating complex feedback loops difficult to capture in traditional frameworks.

Data harmonization is another significant barrier to clinical uptake. Genomic data harmonization involves reconciling different sequencing platforms, analytical pipelines, and annotation standards which can introduce systematic biases if not properly addressed [159]. Clinical data harmonization faces complexities from varying electronic health record systems, coding standards, and documentation practices. Laboratory records may use different units, clinical observations different terminologies, and treatment protocols may vary significantly across institutions. Temporal alignment of multi-modal data presents another significant challenge as genomic data is collected at specific time points while clinical data accumulates continuously, making it difficult to create coherent longitudinal patient profiles for ML training. Patient data is often siloed within healthcare systems due to privacy regulations and competitive concerns. Federated learning approaches offer potential solutions but introduce additional technical complexities [160]. Applying consistent guidelines and principles for data structure harmonization are critical; for example Findable, Accessible, Interoperable, and Reusable (FAIR) principles for data sharing [161] are gaining popularity. There have also been some advances in scalable approaches [162] and guidelines on using ML ethically [163]. However, these need more empirical validation before implementing within health care systems.

## Core principles for best practices

The state of art for best practices in pathogenic variant detection is a rapidly moving target, however core design principles are key in creating a robust and flexible framework able to integrate new modalities as they gain traction in precision medicine. Here we describe several core principles needed to develop a system appropriate for both the current and future needs in precision medicine.

For general design considerations, it is critical to design a workflow that is modular, scalable, parallelisable, secure, reproducible and flexible. A modular design that can incorporate new data types and algorithms as needed is a key requirement when working in this rapidly evolving space. Design flexibility is also key to ensure longevity. For example the ability to run multiple tools and employ a consensus based approach is increasingly being recognised in a variety of applications including variant detection and RNA-Seq data analysis [164]. It is also preferable to incorporate well-tested standardised tools when available to avoid introducing unintentional errors arising from less well tested internally developed software. Another key consideration is the ability to parallelise and scale analysis components, important for reducing turnaround time for individual patients and for handling an increasing volume of samples. Consistency regarding input format requirements and outputs is also critical in ensuring backwards compatibility and the ability for re-analysis.

More specific considerations for precision medicine are around security, interactivity and the ability to generate concise clinical reports. Handling patient data securely is critical for many reasons. Patient data that includes genomic information, medical records and family histories requires the utmost care and sensitivity to meet patient expectations. Misuse of such data may lead to discrimination and potential legal ramifications. De-identification is a common approach however it must be done properly to not allow re-identification via cross referencing of metadata or other public datasets. Ideally de-identification capability needs to be managed via an additional layer of access control. Security needs to be balanced with the development of interactive systems for clinicians who interrogate the data to identify pathogenic variants. Such web-based tools are critical however they need to ensure data is secure and protected throughout. The interface needs to support variant filtering and display variant summaries with the information needed to assess pathogenicity. The interface should support free text entry where clinicians record their determination and describe the evidence justifying the classification. A final consideration is the ability to develop robust clinical reports. Developing user friendly reports requires many iterations with clinicians to determine both the filters to employ and the level of detail to include for each candidate driver. The report design requires flexibility to incorporate addition, often disease specific, information as needed.

Arguably the most critical component in precision medicine system is reproducibility. There are many practical considerations needed to ensure complete reproducibility, which can be achieved through a combination of code versioning, log file generation, robust testing suites and employing a software pipeline manager. Versioning all code and config files during development coupled with thorough logging of all commands lays the foundation for reproducible workflows. Extensive documentation of code and protocols is also critical particularly when multiple team members are involved. The development of a robust testing suite with gold standard datasets will help ensure any changes will not generate unintended downstream consequences. Finally, it is recommended to employ a modern pipeline manager tool such as NextFlow or Snakemake to facilitate running the workflow in a variety of hardware infrastructures including local infrastructure, HPC or cloud. Collectively following these design principles will drastically increase the reproducibility and longevity of the workflow.

## Discussion

**Fig. 2:**
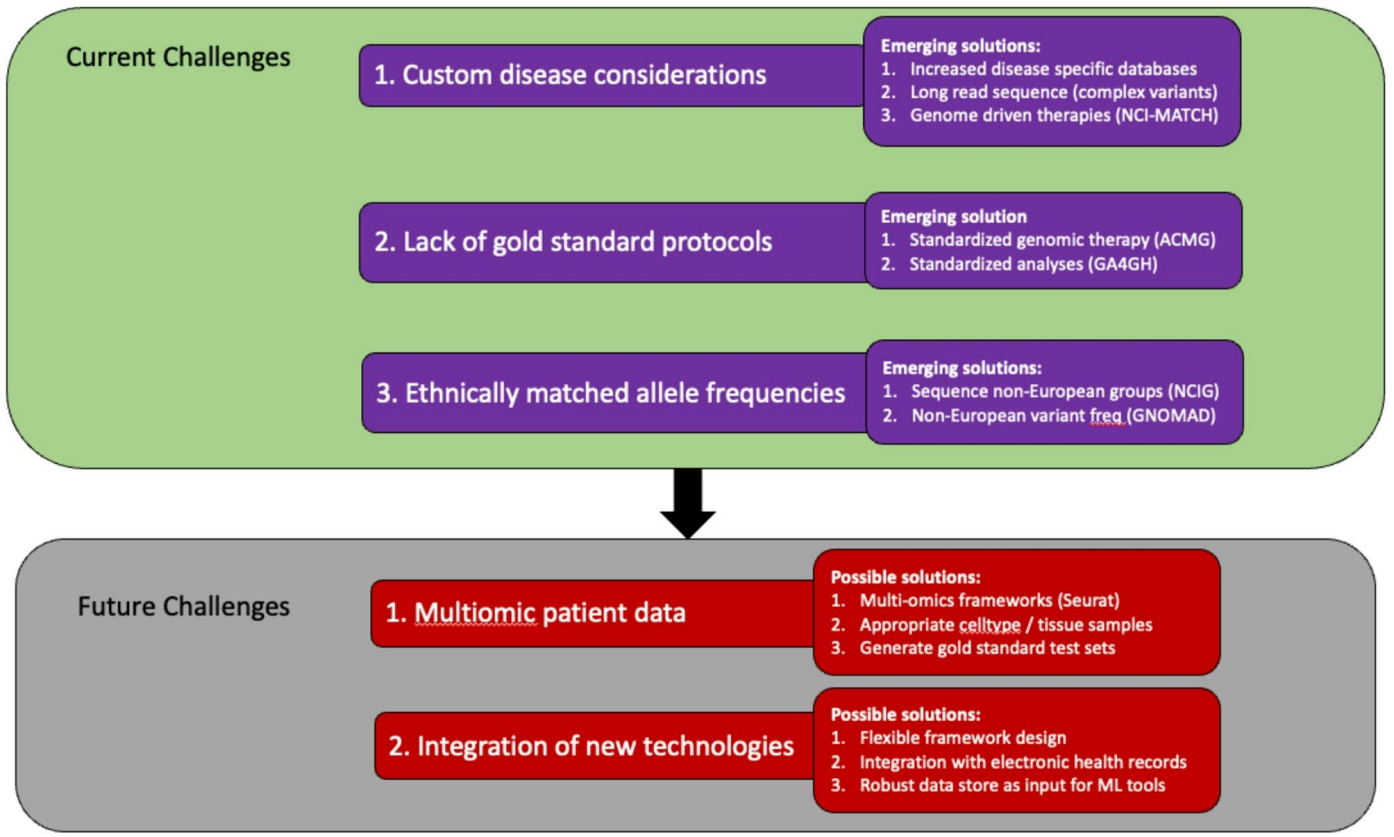
Current and future challenges

While precision medicine has increased diagnosis rates around the world, current challenges exist including handling sensitive patient data and the lack of disease specific annotation, data standards and ethnically matched variant annotations. Future challenges include the need of integration of multi-omic data and the incorporation of new NGS data types (Fig. 2).

### Current challenges

The systematic annotation of genetic variants within precision medicine programs has led to the discovery of large numbers of pathogenic variants however it has become clear no single strategy is universally effective for all diseases. Success within a disease class depends on multiple factors including sequence technology, patient selection and annotation strategy. For example, pedigree sequencing has been critical in identifying disease-causing *de novo* mutations driving neurodevelopmental disorders [165] and autism spectrum disorders [166] and in tracing inheritance patterns across multiple generations in condition like Huntington’s disease [167]. In autoimmune disease research, B-cell or T-cell receptor repertoire sequencing is increasingly utilised to identify pathogenic clonal lineages [168]. Additionally, certain mutation types are strongly associated with a specific disease class such as copy number variations (CNVs) in neurological and autoimmune disorders [19, 169] and splicing variants in Duchenne muscular dystrophy [170]. Careful patient selection based on genomic profiling has been instrumental in matching patients with targeted therapies as demonstrated by studies such as the NCI-MATCH (Molecular Analysis for Therapy Choice) trial [171]. Collectively these findings underscore the need for additional disease-specific considerations to improve genetic diagnosis yields.

A major obstacle to incorporating genomic data into clinical practice is the lack of standard procedures for both analysing NGS data and summarising the relevant information into clinical reports. While progress has been made, consistent and reproducible methods remains essential to ensure the reliability of genomic results [172]. While it is widely acknowledged that ACMG standards and guidelines serve as the de facto gold standard for genetic therapy, their recommended datasets and workflows are often distributed across different online platforms and databases [173]. Further, laboratories often employ different tools and cutoffs, resulting in discrepancies in variant classification with such inconsistencies impacting patient care [174].

Harmonizing interpretation guidelines is crucial for providing clinicians with reliable genomic information to guide personalized medical interventions [175]. However, any standardization must remain flexible given the fast pace of technological advancement and evolving data types [176]. Establishing global collaborations and encouraging data sharing initiatives can contribute to the development of comprehensive, standardized guidelines such as the Global Alliance for Genomics and Health (GA4GH) [177]. Standardized guidelines enhance consistency across laboratories, improve accuracy in variant classification, and ultimately contribute to the reliability of genomic data.

The global genetic landscape is shaped by ethnic diversity and influenced by historical, geographical, and demographic factors [178]. To account for this, it is important to establish population-specific databases to capture the unique genetic variations across diverse ethnic groups. GNOMAD, for instance, has been instrumental in cataloguing genetic variations across varied populations, offering a valuable resource for ethnicity level variant frequency estimations [179]. This information can influence not only disease susceptibility but treatment response; for example, certain pharmacogenetic variants affect drug metabolism differently in various populations [180]. However establishing population-specific databases comes with challenges, including ethical considerations, data privacy, and ensuring adequate representation [181]. Initiatives such as ‘All of Us Research Program’ aims to address these issues by building inclusive, large-scale dataset that reflects the global genetic diversity. Such efforts are crucial in unravelling the complexities of genetic variants across distinct ethnic groups.

### Future challenges

The rise of affordable sequencing technology has led to a growing reliance on data generated across various biological levels [182]. For example, the microbiome is increasingly being linked to human health outcomes with composition shifts observed during the onset of many diseases such as type II diabetes [183–186]. Integrating metagenomic and other multi-omic patient data with clinical information has the potential to improve prognostics and predictive accuracy of disease phenotypes ultimately leading to better treatment and prevention strategies [187, 188]. However, the analysis of these complex datasets remains a challenge due to the inherent heterogeneity in individual omics datasets and the computational resources required for analysis and integration [182]. The rapid pace of change within the multi-omics space means benchmarking studies are essential to ensure appropriate tools are chosen to address specific biological questions [189–191]. Future developments should prioritise reducing complexity, enhancing interoperability, and creating user-friendly frameworks to consolidate multi-omics data.

The effective application of precision medicine depends on precise, evidence-driven interpretation of genetic data, ensuring proper clinical management and care, while avoiding flawed conclusions that could lead to harm [192]. Genomic data is inherently dynamic and influenced by fast moving technological advancements meaning variants that were once classified as benign may need re-evaluation as new evidence emerges [193]. Advanced informatics and ML algorithms are poised to enable real-time data integration of diverse datasets, identifying clinically-relevant patterns that contribute to the continuous refinement of variant interpretation [194]. Such systems will empower clinicians with the most up-to-date variant interpretations, enhancing the precision and effectiveness of personalized healthcare. Central to this vision is working with Electronic Health Records (EHRs), which serve as comprehensive repositories of patient-specific data, encompassing medical histories, treatment responses, and other relevant information. Incorporating EHR data into the genetic variant prioritisation process enables a holistic view of the patient’s health journey [195]. Embracing a patient-centric framework will allow healthcare providers to tailor genetic interpretations that align with individual needs, ultimately realising the potential of personalised patient care.

## Conclusion

Prioritising disease-causing genetic variants is fundamental for progressing personalized medicine, improving clinical diagnostics, and understanding genetic contributions to diseases. The adoption of precision medicine programs underscores the importance of prioritizing genetic variants, tailoring patient care based on genetic makeup and individual characteristics. While the affordability of quality sequence data has improved, substantiating the link between genes and diseases remains resource intensive. Accurate identification of disease-causing variants enhances diagnostic precision, aiding in early detection and targeted interventions for genetic disorders. Addressing current challenges today will ensure better precision medicine in the future.

## Data Availability

No datasets were generated or analysed during the current study.
